# Quantification Beyond Binary of MR FLAIR Hyperintensity Lesions in Acute Ischemic Stroke of Unknown Time Since Onset

**DOI:** 10.3390/diagnostics16111641

**Published:** 2026-05-27

**Authors:** Cecilie Juul Mørck Offersen, Jacob Johansen, Kaining Sheng, Andreas Hjelm Brandt, Thomas Clement Truelsen, Akshay Pai, Michael Bachmann Nielsen, Jonathan Frederik Carlsen

**Affiliations:** 1 Department of Diagnostic Radiology, Copenhagen University Hospital, Rigshospitalet, 2100 Copenhagen, Denmark; kaining.sheng@gmail.com (K.S.); andreas.hjelm.brandt.02@regionh.dk (A.H.B.); ap@cerebriu.com (A.P.); mbn@dadlnet.dk (M.B.N.); jonathan.frederik.carlsen@regionh.dk (J.F.C.); 2 Department of Computer Science, University of Copenhagen, 2100 Copenhagen, Denmark; jj@di.ku.dk; 3 Department of Neurology, Copenhagen University Hospital, Rigshospitalet, 2100 Copenhagen, Denmark; thomas.clement.truelsen@regionh.dk; 4 Department of Clinical Medicine, University of Copenhagen, 2100 Copenhagen, Denmark; 5 Cerebriu A/S, 1434 Copenhagen, Denmark

**Keywords:** automated assessment, FLAIR quantification, FLAIR hyperintensity, DWI-FLAIR mismatch, acute ischemic stroke, wake-up stroke, NIHSS, thrombolysis

## Abstract

**Background:** High inter-rater variability in DWI-FLAIR mismatch assessments for acute ischemic stroke (AIS) has spurred interest in assisting automated imaging measures. In this study, we explored whether DWI-FLAIR mismatch assessment and automated quantification of FLAIR hyperintensity, DWI-FLAIR volume ratio, and DWI volume could predict changes in NIHSS score following intravenous thrombolysis (IVT) in patients with AIS of unknown time since onset (TSO). We also exploratively compared radiological DWI-FLAIR mismatch assessments and imaging measures between patients who received IVT and those who did not. **Methods:** We conducted a retrospective, exploratory, single-center study analyzing brain MRIs from a consecutive cohort of patients with suspected AIS and unknown TSO admitted over two years. Patients with DWI hyperintensity lesions related to ischemia, identified automatically and subsequently verified radiologically, were included. We examined the correlation between automated imaging measures, retrospective DWI-FLAIR mismatch assessments, and changes in NIHSS score from baseline to 24 h post-treatment. **Results:** Of 333 patients included, 109 received IVT (mean age 68.9 ± 15.2 years) and 224 did not (mean age 70.8 ± 13.8 years). The median baseline NIHSS score was 5 in both groups, improving to 2 after IVT. The DWI volume significantly correlated with changes in NIHSS score (*p* = 0.002); FLAIR intensity demonstrated borderline significance (*p* = 0.056); and DWI-FLAIR volume ratio showed no statistically significant association in this cohort (*p* = 0.511). We did not find statistical evidence that the retrospective binary mismatch assessment was correlated with differences in outcome (*p* > 0.145). **Conclusions:** This study supports moving beyond binary DWI-FLAIR mismatch, suggesting that continuous, automated imaging parameters could potentially assist the radiologist in AIS management. As evidence remains preliminary, large-scale research is needed to establish clinical utility.

## 1. Introduction

Observational studies have reported that approximately 25% of all patients with acute ischemic stroke (AIS) have an unknown time since onset (TSO), either because it was unwitnessed or occurred as a “wake-up stroke” (WUS) [1,2,3]. Early management and decision of treatment with intravenous thrombolysis (IVT) in patients with AIS and unknown TSO relies on the radiological assessment of magnetic resonance Diffusion-Weighted Imaging—T2 Fluid-Attenuated Inversion Recovery (DWI-FLAIR) mismatch [3,4]. Patients with an absolute mismatch are eligible for treatment with IVT [5]. In “WAKE-UP”, a randomized clinical trial, and a multicenter observational study, IVT was shown to be beneficial for AIS patients with unknown TSO and identified DWI-FLAIR mismatch, showing better functional outcome compared to patients with DWI-FLAIR mismatch, who did not receive IVT [3,6]. However, the radiological mismatch assessment is susceptible to discrepancies due to the individual interpretation by the radiologist of the binary DWI-FLAIR mismatch assessment. This introduces variability and the risk of excluding eligible patients [1,7,8].

We identified two studies on the use of non-binary DWI-FLAIR mismatch assessment [9,10]. In one study using a modified DWI-FLAIR assessment comprising three categories—clear mismatch, partial mismatch, and no mismatch—a comparable outcome was found between patients retrospectively assessed as having a clear mismatch and those assessed as having a partial mismatch. Partial mismatch is explained by the MRI showing some degree of FLAIR hyperintensity within the area of the ischemic lesion [9].

Building on these findings, the observation of similar outcomes between clear and partial mismatch groups suggests that the traditional binary DWI-FLAIR mismatch classification may not fully capture the nuances relevant to patient prognosis. This raises important questions about the predictive value of the binary mismatch approach and highlights the potential of quantitative methods. Therefore, in addressing the current gap in understanding whether DWI-FLAIR mismatch assessment relates to treatment outcomes, our study not only explores the prognostic value of the conventional binary mismatch classification but also whether automated, objective measurements, specifically the DWI-FLAIR lesion volume ratio and quantitative FLAIR intensity measures from a presented algorithm [11,12], and the DWI volume, can provide additional prognostic information. By doing so, we aim to explore potential imaging biomarkers that could refine eligibility criteria and improve patient selection for intravenous thrombolysis.

## 2. Materials and Methods

### 2.1. Inclusion of Patients

The patients included in this study were chosen from a consecutive cohort of patients admitted under suspicion of AIS and unknown TSO, in the period between March 2019 and September 2021. All patients were aged 18 years or more. All patients had a brain MRI upon admission.

The inclusion criteria comprised an available brain MRI with DWI, ADC, and FLAIR sequences performed upon hospital admission due to AIS and unknown onset; age above 18 years; and automated detection of an intracranial DWI lesion.

IVT treatment response was based on routine clinical data at baseline and at 24 h after IVT administration, assessed using the change in the reported National Institutes of Health Stroke Scale (NIHSS) [13].

The study was approved by the Danish National Center for Ethics.

### 2.2. Exclusion Criteria

To assess potential outcome prediction from DWI-FLAIR lesion volume ratio and quantitative FLAIR intensity measures, we used the output of a recently evaluated Volume and Intensity FLAIR Algorithm (VIFA) [11] and a DWI segmentation tool (Apollo v.2.1, developed by Cerebriu A/S, Copenhagen, Denmark). Patients were excluded if the DWI segmentation model did not detect a DWI lesion, as the automated measures from the VIFA only work if a DWI lesion is detected.

Afterwards, all MRIs were radiologically assessed and excluded if the DWI lesion was unrelated to an acute ischemic lesion, e.g., tumors, hemorrhage, artifacts, and other pathologies. Two assessors had to agree on the presence of a lesion related to ischemia; otherwise, the patient was excluded from the study.

### 2.3. The Automated Screening

All patients’ baseline MRIs were assessed with automated screening. Identification of patients with MR DWI-positive lesions related to ischemia was based on automated detection by the DWI segmentation tool (Apollo v.2.1, Cerebriu A/S) using a U-net model [14]. The DWI lesion volume alone was investigated as an independent predictor of NIHSS score change.

### 2.4. The Radiological Assessment

All the magnetic resonance images were additionally assessed by two radiologists for the identification of DWI lesions compatible with an ischemic lesion.

The assessments were performed by two neuro-radiologists, each with >10 years of clinical experience, and one radiology resident with 4 years of clinical experience. All three assessors were blinded to the original radiology report and clinical data. They assessed the examinations identified by the DWI segmentation tool. ADC, DWI and FLAIR sequences were available.

The cohort was divided into three numerically equal batches. Each radiologist independently assessed two out of three batches, ensuring that two radiologists evaluated each examination, determining whether the scan showed DWI-FLAIR mismatch or no mismatch.

This resulted in three groups:

1. Agreement of DWI-FLAIR mismatch;
2. Disagreement of DWI-FLAIR mismatch;
3. Agreement on no DWI-FLAIR mismatch.

**Figure 1:**
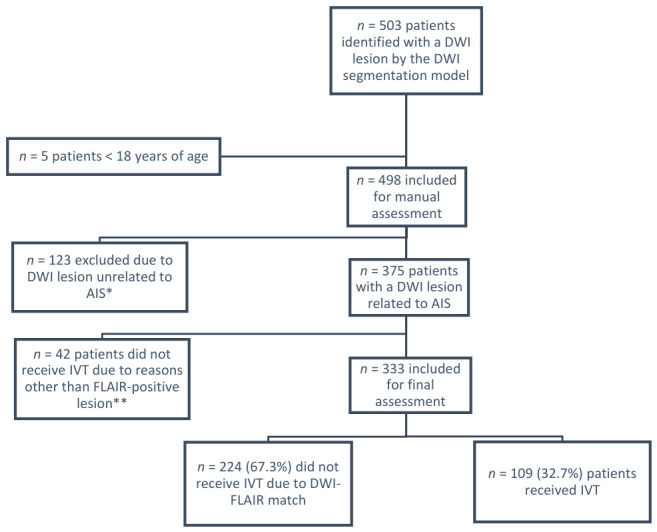
Inclusion/exclusion flowchart. * Unrelated to AIS, e.g., hemorrhagic lesion, non-acute stroke, tumor, artefact, and other pathologies. ** Other causes of exclusion than FLAIR positive lesions included patients described with multiple microbleeds, hemorrhage, tumors, lesions compatible with multiple sclerosis or postictal oedema. Only patients with radiological agreement of AIS lesion on MRI between two radiologists were included.

Subsequently, we divided the cohort into two groups: those who received IVT and those who did not. A flowchart of the inclusion/exclusion process is shown in Figure 1.

### 2.5. Automated FLAIR Lesion Measurements from VIFA

The automated measurements from the VIFA [11] segments FLAIR hyperintensities using three main features:

1. A region of interest, defined by projecting the visible ischemic lesion from DWI onto the FLAIR image.
2. The ratio of FLAIR intensity within this region to that of a reference region on the contralateral side.
3. The mean and standard deviation of FLAIR intensities across the brain, excluding the region of interest and the ventricles.

The two measurements obtained from the VIFA are: 1. the volume ratio between the DWI and FLAIR sequence segmentations (ranging from 0 to 1); 2. the intensity of the FLAIR lesion [11,12]. The VIFA also provides an assessment of mismatch or no mismatch and a segmentation of the lesion, making it visually verifiable. This visual identification of the lesion helps identify and exclude artifacts, e.g., potential distortion and high signal intensity near the skull base. This calculation of high signal intensity volumes on both DWI and FLAIR is performed in real time, taking less than one minute, underscoring the method’s practicality and potential for rapid clinical implementation.

### 2.6. Comparison of IVT vs. Non-IVT Patients

The VIFA measurements identified for patients receiving IVT (DWI-FLAIR mismatch) were compared to those who were assessed as ineligible (DWI-FLAIR no mismatch) upon admission. For all patients not receiving thrombolysis, the cause of exclusion was identified using the description in the electronic patient files and the associated radiological report. Patients excluded from thrombolysis for causes other than FLAIR-positive lesions were excluded from the final comparison. This could include patients described with multiple microbleeds, hemorrhage, tumors, and lesions compatible with those observed in multiple sclerosis or postictal edema. Patients with a large amount of chronic white matter hyperintensities (WMHs) were included if the hyperintensities were not classified as acute FLAIR-positive lesions in the radiological report.

### 2.7. Clinical Data

Clinical data of all included patients comprised age, gender, and NIHSS score for assessment of stroke severity [4,13], as well as cardiovascular comorbidities. For patients receiving IVT, both baseline NIHSS scores and, when available, 24 h post-treatment NIHSS scores were obtained. When available, the Modified Rankin Scale (mRS) score at 90 days was obtained. Because mRS data were incomplete and likely skewed toward patients with better outcomes, we selected NIHSS score change as the primary outcome to provide a more comprehensive and unbiased assessment.

Incidence of symptomatic intracranial hemorrhage with an increase in NIHSS score of ≥4 in both the IVT and non-IVT groups within the first 24 h after IVT treatment or after hospital admission, respectively, was also registered.

### 2.8. Statistics

We employed a Generalized Linear Model (GLM) to investigate the relationship between quantitative imaging biomarkers and short-term neurological improvement following intravenous thrombolysis (IVT). Specifically, the regression analysis included FLAIR intensity, DWI-FLAIR volume ratio, and the DWI lesion volume relative to total brain tissue, calculated by dividing the segmented DWI lesion area by the area of the skull-stripped brain (excluding ventricles). The baseline NIHSS score was also included as a covariate in the model to adjust for initial stroke severity. The primary outcome variable was the change in NIHSS score from baseline to 24 h after IVT administration, which serves as a measure of early neurological response to treatment.

The purpose of the GLM regression analysis was to determine the independent association of each imaging parameter with NIHSS score change, adjusting for the influence of the other variables in the model. Statistical significance was set at a P value of 0.05. To ensure the validity of the regression model and to assess potential multicollinearity among predictor variables, we calculated the Variance Inflation Factor (VIF) and performed a Variance Decomposition Proportions (VDP) analysis.

Additionally, we compared the three groups defined by manual radiological assessment—agreement of mismatch, disagreement, and agreement of no mismatch—using the Kruskal–Wallis test. This non-parametric test allowed us to evaluate whether the extent of NIHSS score improvement differed significantly among patients grouped by their visual DWI-FLAIR mismatch status after receiving IVT.

This comprehensive approach enabled us to explore both the independent and group-based effects of quantitative imaging biomarkers and visual mismatch categories on early clinical outcomes, while checking for statistical assumptions and potential confounding effects.

## 3. Results

### 3.1. Cohort Characteristics

The automated and radiological detection process resulted in the inclusion of 333 patients for final assessment. This included 109 patients who received IVT, with a mean age of 68.9 ± 15.3 years, of whom 51 (46.8%) were female, as shown in Table 1. The group of patients who did not receive IVT comprised 224 patients, with a mean age of 70.7 ± 13.79 years, of whom 105 (46.9%) were female.

### 3.2. Radiological Assessment and Relation to NIHSS Score Change

Inter-rater agreement for DWI-FLAIR mismatch assessments was evaluated in a cohort of 333 patients. Using the dichotomized mismatch versus no mismatch, the observed agreement was 76.9%, with a Cohen’s kappa of 0.48 (95% CI: 0.38 to 0.58), reflecting moderate agreement. Each case was evaluated by two of three radiologists, with rater pairs varying across batches (see Supplementary Table S1).

The median baseline NIHSS score for patients treated with IVT was 5 (±SD 4.47, range 0–22). The median NIHSS score post-treatment was 2 (±SD 4.08, range 0–14). The baseline median NIHSS score in the group of patients that did not receive IVT was 5 (±SD 5.2, range 0–29). NIHSS data were unavailable for 19 out of the 109 IVT patients and for 49 out of the 224 non-IVT patients. Table 2 shows the median, range and change in NIHSS score for all three radiological assessment groups.

The median NIHSS score improved in all three mismatch groups of patients who received thrombolysis. Nine patients were registered with an increase in post-treatment NIHSS score ranging from a minimum of 1 to a maximum of 7. They were distributed between the three assessment groups, as shown in Table 2. We found no statistically significant difference in outcome between the three assessment groups (*p* > 0.05).

### 3.3. Outcomes

All IVT patients received a follow-up CT 24 h post-treatment, following standard procedure at the hospital. Two IVT patients (1.8%) suffered from symptomatic intracranial hemorrhage within 24 h post-treatment [16]. One of them received both IVT and endovascular thrombectomy (EVT). Two other patients had radiological signs of minor parenchymatous hemorrhage, and two had minimal hemorrhage transformation within the lesion on the 24 h follow-up CT scan. However, all of them showed improvement in NIHSS score post-treatment and regression of hemorrhage on later follow-up CT scans.

Five patients (2.2%) in the group of 224 individuals that did not receive thrombolysis had a hemorrhagic transformation and were affected by an increase in NIHSS score. The 90-day mRS score was available for only 61.5% of the included patients, with 10 patients deceased before day 90. Among those with a registered mRS score, the majority (71.6%) had a score of 0 or 1, and five patients had a pre-treatment mRS score ≥ 2.

### 3.4. FLAIR Quantification Including Comparison of IVT vs. Non-IVT Patients

**Figure 2:**
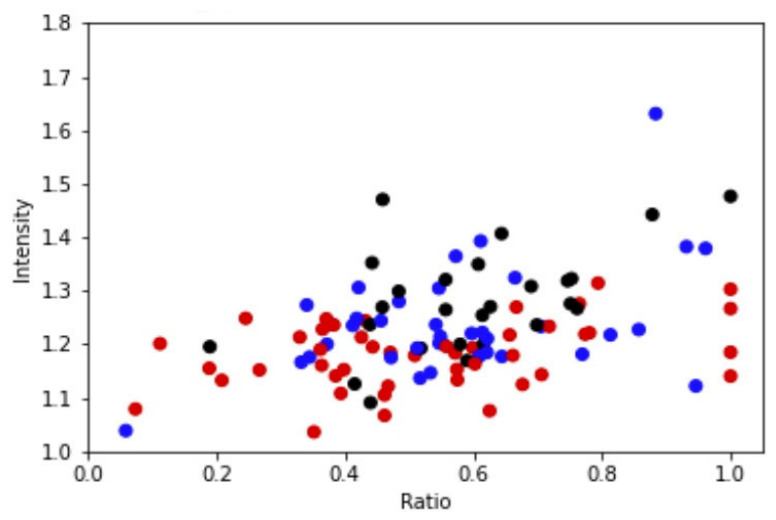
Distribution of the relative FLAIR intensity and DWI-FLAIR ratio for all 109 patients receiving thrombolysis, showing the blinded radiological mismatch classification. Mismatch: red; disagreement: blue; no mismatch: black. The comparison between groups is purely descriptive and should not be interpreted as a formal group comparison.

In the comparison of IVT vs. non-IVT patients, we found a noticeable overlap of patients between the two groups, who had similar automatic measures of intensity and DWI-FLAIR ratios. Group comparisons are descriptive and were not formally analyzed. In Figure 2, all patients who received IVT are shown and divided by color into three groups formed by the radiological mismatch assessment. We found no statistical evidence that the assessment for either of the three groups was predictive of outcomes.

**Figure 3:**
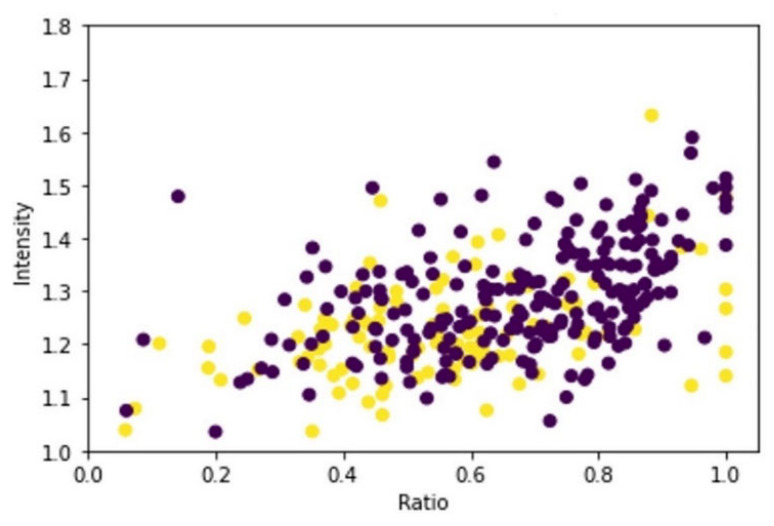
Distribution of FLAIR intensity and DWI-FLAIR volume ratio for all 333 included patients. IVT patients: yellow; non-IVT patients: purple. The comparison between groups is purely descriptive and should not be interpreted as a formal group comparison.

Figure 3 shows all included patients divided into two groups: IVT and non-IVT.

DWI volume demonstrated a significant correlation with change in NIHSS score (*p* = 0.002). In this cohort, a large absolute change in NIHSS score did not necessarily indicate a proportionally large clinical difference across patients.

FLAIR intensity demonstrated a borderline significant correlation (*p* =0.056), whereas no statistically significant association was observed for DWI-FLAIR volume in this cohort (*p* = 0.511). Baseline NIHSS score was significantly associated with the outcome (*p* < 0.001). We did not find statistical evidence that the retrospective mismatch assessment was correlated with differences in outcome (*p* = 0.145).

**Figure 4:**
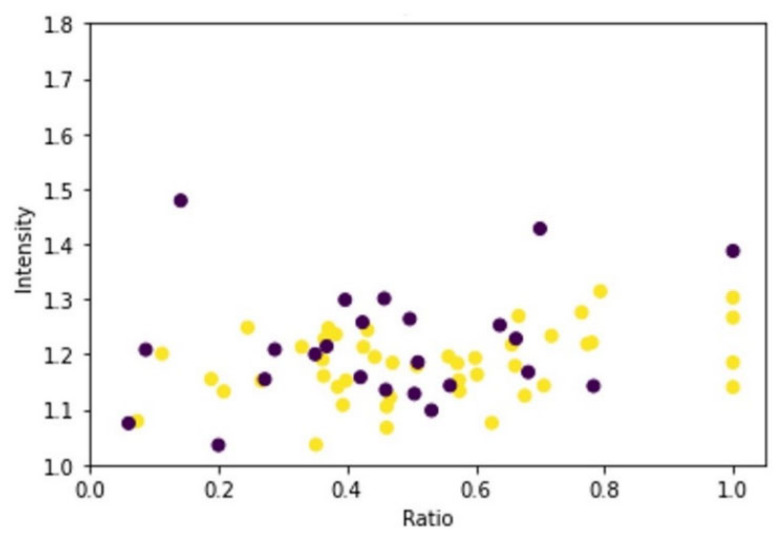
Distribution of automated mismatch quantification for IVT-treated (yellow) and non-IVT-treated (purple) patients, including all patients manually classified as mismatch patients in the retrospective assessment. The comparison between groups is purely descriptive and should not be interpreted as a formal group comparison.

VIFA measurements for IVT and non-IVT patients retrospectively assessed as mismatches are presented in Figure 4, showing a considerable overlap between the two groups in the objective measurements.

We assessed risk of collinearity using the Variance Inflation Factor (VIF) and Variance Decomposition Proportions (VDP). The analysis showed a low risk of collinearity. The results are shown in Appendix A.

## 4. Discussion

Through automatic FLAIR quantification, the FLAIR intensity demonstrated a borderline correlation with NIHSS change (*p* = 0.056), which may be attributable to limited statistical power rather than a true absence of association. This interpretation is further supported by the performed VIF and VDP, shown in Appendix A.

In this cohort, we did not observe a statistically significant association between the DWI-FLAIR volume ratio and NIHSS change (*p* = 0.511). Nevertheless, a deeper understanding of the relationship remains important. Further investigation with larger cohorts could help clarify this relationship. If a correlation is established, it would enhance the objectivity and reproducibility of mismatch evaluation, especially in situations where visual assessments are difficult. Conversely, if no correlation is found, this would also be informative for refining assessment strategies in clinical practice.

We found that both baseline NIHSS (*p* < 0.001) and DWI volume demonstrated a significant correlation with change in NIHSS score (*p* = 0.002). Our findings of the correlation of DWI volume and prediction of NIHSS score change correspond well to the results of a multivariate analysis on the DWI volume as a predictor of outcome [17].

The blinded, retrospective manual assessment of DWI-FLAIR mismatch led to no statistical evidence that the manual mismatch assessment could be used as a predictor of NIHSS score change. This aligns with the findings of earlier studies, where patients with a complete mismatch and patients with any mismatch had comparable and beneficial outcomes from treatment with IVT [18]. Moreover, in our study, all three groups, i.e., agreement of mismatch, disagreement, and agreement of no mismatch, had improvement in the 24 h post-treatment median NIHSS score. A survey of mismatch assessments in AIS patients showed comparable outcomes in patients assessed as having negative FLAIR images and those with visual FLAIR signals, but still some degree of mismatch [10]. Likewise, another study on thrombolysis in wake-up stroke (WUS) using the MRI mismatch concept and a retrospective radiological assessment found that the groups with clear mismatch and those with some degree of FLAIR lesion visibility were comparable in outcome performance [9]. 

A safety measure in the present study was the registration of ICH incidence, and we found no increased rate of symptomatic ICH in the IVT group compared to the non-IVT group. This finding is particularly relevant in the context of recent studies that have investigated expanding the treatment window for thrombolysis, including both patients with known and unknown TSO [16,18,19]. The results from a multicenter randomized placebo-controlled trial using perfusion imaging on AIS showed promising potential in treatment for up to 9 h since stroke onset [19]. They found a higher percentage of patients with no or minor neurologic deficits than in the placebo group [19]. In addition, a study compared the severity and outcome in patients with WUS and morning strokes where TSO was known [20]. They found the groups to have comparable characteristics. However, patients with known TSO were more likely to receive IVT than patients with an unknown onset. This could partly be because of safety measures. Clinicians may opt for more conservative approaches due to concerns about the risk of hemorrhage, as an increased risk of intracranial hemorrhage (ICH) has been observed in patients receiving IVT compared to those who did not [6].

However, in the systematic review and multi-analysis by Thomalla et al., no difference in the occurrence of ICH was observed in patients with known TSO compared to patients with unknown TSO [6]. They also concluded that, although a higher mortality rate was observed in IVT patients, the benefit of IVT on functional outcome was evident [6].

### 4.1. Limitations

This exploratory, single-center, retrospective study has a relatively small cohort. The borderline significance for FLAIR intensity (*p* = 0.056) may be attributable to limited statistical power. Incomplete follow-up and absent mRS data in nearly 40% of IVT-treated patients introduce a risk of bias; consequently, mRS was excluded as an outcome, and only patients with a registered NIHSS score were included in the statistical analysis.

The median NIHSS score of 5 reflects a predominantly minor-to-moderate stroke cohort, potentially limiting generalizability to severe cases. Missing follow-up data preclude conclusions on long-term outcomes based on automated measures or mismatch classifications. Additionally, the DWI segmentation model may not capture all ischemic lesions [21,22]. This limits the clinical applicability of the full automated segmentation method. However, our primary aim in this study was not to assess the overall performance of the lesion detection tool, but to explore whether imaging biomarkers extracted through our automated pipeline can serve as predictors of outcome. Therefore, we focused on patients with detectable lesions, and the exact number of exclusions due to failed segmentation does not affect the interpretation of our results.

Finally, the overlap in the automated mismatch quantification between IVT-treated and non-IVT-treated patients in Figure 4 is a descriptive, hypothesis-generating observation; no formal group comparison was performed. A prospective design with pre-specified group comparisons would be required to formally evaluate this relationship.

### 4.2. Perspectives for Future Studies

This exploratory study, which, to our knowledge, is the first to investigate these specific automated imaging measures in relation to early neurological changes following IVT in AIS patients with unknown time of onset, should be viewed as an initial step toward understanding the clinical utility of these measures.

Among the patients who did not receive IVT, we identified a group of patients who had FLAIR intensity and DWI-FLAIR volume ratios comparable to those of the patients who received IVT. Retrospectively, they were found to be in all three radiological assessment groups. This observation suggests that there may be potential value in considering IVT for a broader group of patients, as their imaging markers appear relatively similar, despite current variations in how radiologists assess mismatch.

This emphasizes the importance of further research focused on the outcomes of patients receiving IVT, despite discrepancies in radiological assessments of FLAIR lesion visibility, and with consideration of automated measurements for more standardized treatment selection.

A prospective study with a larger cohort and follow-up data for both patients receiving IVT and those not receiving IVT could provide an enhanced comparison of outcomes between the two groups. In a prospective study identifying the impact on outcome, including other factors, e.g., multiple microbleeds, FLAIR Vascular Hyperintensities, and other white matter hyperintensities, would also be beneficial [23,24].

The findings of this study highlight the potential value of a more individualized approach to outcome prediction and provide further empirical support for continued investigation into the role of advanced imaging biomarkers in clinical decision-making.

## 5. Conclusions

In conclusion, the integration of automated image-based measurements, such as FLAIR lesion quantification, represents a promising step forward in the management of acute ischemic stroke, particularly in cases with unknown time of onset. Our results suggest that moving beyond binary DWI-FLAIR mismatch to incorporate continuous, automated imaging parameters may offer more nuanced and objective support for clinical decision-making. However, as this evidence is preliminary and exploratory, these approaches should be viewed as emerging options. Large-scale studies are needed to determine their clinical utility and to establish their role in the future management of AIS with unknown TSO.

## Figures and Tables

**Table 1:** Clinical characteristics of the included patients.

Patient Characteristics	IVT	No IVT
Number of patients	109	224
Age (mean ± SD)	68.9 ± 15.24	70.8 ± 13.8
Female, *n* (%)	51 (46.8%)	105 (46.9%)
Male, *n* (%)	58 (53.2%)	119 (53.1%)
Hypertension	65 (59.6%)	98 (43.8%)
Hyper cholesterol	52 (47.8%)	77 (34.4%)
AF	5 (4.6%)	18 (8%)
Previous stroke or TCI	16 (14.7%)	29 (12.9%)
DM2	11 (10%)	31 (13.8%)
Currently smoking	8 (7.3%)	28 (12.5%)

TCI: Transient Cerebral Ischemia; DM2: Type 2 Diabetes Mellitus; AF: Atrial Fibrillation.

**Table 2:** Overview of patient treatment response.

NIHSS Score Development in IVT Patients Grouped by Radiological Mismatch Assessment (Baseline NIHSS Score *n* = 109; 24 h NIHSS Score Available for *n* = 90)			
Radiological Agreement	Mismatch	Disagreement	No Mismatch
*n*	46	37	26
Patients with a registered increase in 24 h NIHSS score, with an increase between 1 and 7 (*n*)	2	3	4
Baseline NIHSS score, median	6	4	6
24 h NIHSS score (*n* = 90), median	2	2	4
Overall baseline NIHSS score (all IVT patients, *n* = 109), median, range, SD	5 (range 0–22/SD ± 4.47).		
Overall 24 h NIHSS score (patients with available data, *n* = 90), median, range, SD	2 (range 0–14/SD ± 4.08).		
Safety outcomes			
Symptomatic intracranial hemorrhage *	1	1	0
Death within 90 days after intervention	2	2	2

Baseline NIHSS: NIHSS score before IVT treatment; 24 h NIHSS: NIHSS score 24 h after IVT; mRS: Modified Rankin Scale * symptomatic intracranial hemorrhage within 24 h post-treatment with an increase of ≥4 in NIHSS score [15].

## Data Availability

The datasets presented in this article are not readily available due to privacy limitations.

## Supplementary Materials

The following supporting information can be downloaded at: https://www.mdpi.com/article/10.3390/diagnostics16111641/s1, Table S1: Inter-rater agreement (Cohen’s kappa) for DWI–FLAIR mismatch assessment (*n* = 333). Fi–now listed.

## Abbreviations

The following abbreviations are used in this manuscript:

AIS	Acute Ischemic Stroke
DWI	Magnetic Resonance Diffusion-Weighted Imaging
DWI-FLAIR	Magnetic Resonance Diffusion-Weighted Imaging–T2 Fluid-Attenuated Inversion Recovery
FLAIR	T2 Fluid-Attenuated Inversion Recovery
FVH	FLAIR Vascular Hyperintensity
IVT	Intravenous Thrombolysis
MRI	Magnetic Resonance Imaging
r-tPA	Recombinant Tissue Plasminogen Activator
TSO	Time Since Onset
WUS	Wake-Up Stroke
