# Supplementary material for: Quantification Beyond Binary of MR FLAIR Hyperintensity Lesions in Acute Ischemic Stroke of Unknown Time Since Onset
Source: Diagnostics (Basel). 2026 May 27;16(11):1641. doi: 10.3390/diagnostics16111641 (PMC13257346; doi:10.3390/diagnostics16111641)
Supplement: Supplementary file 1 [file diagnostics-16-01641-s001.zip › diagnostics-4260448-supplementary.pdf]

Supplementary Materials

Supplementary Table S1. Inter-rater agreement (Cohen´s kappa) between two independent radiological readings of DWI–FLAIR mismatch in the full assessed cohort (n = 333).

Classification	N	Observed agreement (%)	Cohen's κ	95% CI	Strength of agreement
Binary (mismatch / no-mismatch)	333	76.9	0.48	0.38 to 0.58	Moderate

Dichotomised assessment of mismatch vs no-mismatch. Each examination was rated by two of three radiologists; the rater pair varied across batches. CI, confidence interval; DWI, diffusion-weighted imaging; FLAIR, T2-fluid-attenuated inversion recovery; Cohen´s k, Cohen's kappa.
